# Preferences and Perspectives of Black Male Barbershop Patrons on Receiving Health Care in Nontraditional Settings

**DOI:** 10.1089/heq.2023.0157

**Published:** 2023-12-12

**Authors:** Sarah Chong, Brittany Huynh, Stephanie Wong, Temesgen Woldeyesus, Melvin Faulks, Kenneth El-Amin, Jabari Thibeaux, Joseph Lewis, Robert Harlin, Mario Carter, Ramy Shatara, Crystal Zhou, Akinyemi Oni-Orisan

**Affiliations:** ^1^ Department of Clinical Pharmacy, University of Maryland, Baltimore, Maryland, USA.; ^2^ Department of Clinical Pharmacy, University of California San Francisco, San Francisco, California, USA.; ^3^ Department of Medicine, Stanford University, Palo Alto, California, USA.; ^4^ Department of Clinical Programs, Roots Community Health Center, Oakland, California, USA.; ^5^ Chicago 2 Barbershop, San Francisco, California, USA.; ^6^ Institute for Human Genetics, University of California San Francisco, San Francisco, California, USA.; ^7^ Department of Bioengineering and Therapeutic Sciences, University of California San Francisco, San Francisco, California, USA.

**Keywords:** health care access, primary care, chronic diseases, pharmacy, delivery of health care, community health services

## Abstract

**Introduction:**

Non-Hispanic Black men experience a disproportionate rate of morbidity and mortality from hypertension, cardiovascular disease, and other chronic conditions in the United States. Studies have demonstrated the efficacy of community-based health outreach in settings not traditionally utilized for health care. Understanding how potential future participants view health care services in nontraditional settings is a necessary step to ascertain the success of these interventions in the real world. Our study objective was to explore the preferences of Black male barbershop patrons regarding health care-provided services in these nontraditional settings.

**Methods:**

We recruited patrons of a Black-owned barbershop in the San Francisco Bay Area. Study participants were asked to complete a survey assessing individual attitudes and preferences toward the idea of receiving health care services in traditional and nontraditional settings.

**Results:**

Among non-Hispanic Black males (*n*=17), 81% agreed or strongly agreed that they would prefer to receive health care in traditional clinics. Receiving care at the pharmacy (56% agreed or strongly agreed) and the patient's own home (53% agreed or strongly agreed) were the next most preferred locations. A minority of participants agreed or strongly agreed that they preferred to receive health care in nontraditional settings: 47% for barbershops, 19% for churches, and 6% for grocery stores.

**Discussion:**

Participants expressed preference for traditional over nontraditional settings, despite listing barriers that may be addressed, in part, by nontraditional settings. One potential reason for this is simply a lack of familiarity. Establishing and normalizing nontraditional clinical settings may allow for enhanced acceptance within Black communities, ultimately increasing health care access.

## Introduction

Non-Hispanic Black (herein referred to as Black) people experience a disproportionate rate of poor outcomes from preventable chronic diseases such as diabetes,^1,2^ atherosclerotic cardiovascular disease,^3,4^ and hypertension,^5–7^ trends which have only worsened over the past two decades.^8,9^ New community-based approaches for the improved screening and management of chronic diseases for Black communities are necessary to address these disparities.

In response to this need, efforts to explore the effectiveness of health interventions have been conducted in barbershops,^9–12^ churches,^13,14^ and other nontraditional health care settings.^15–18^ A landmark cluster-randomized trial for Black men with uncontrolled hypertension (the Los Angeles Barbershop Blood Pressure Study [LABBPS]) investigated the impact of novel interventions for blood pressure management in the setting of Black-owned barbershops.^18^ Health promotion by trained barbers to barbershop patrons resulted in clinically relevant blood pressure reduction in both treatment arms, demonstrating the potential of providing patient care in settings not traditionally utilized for health care interventions. More importantly, blood pressure reduction showed the greatest success in the “treatment” arm (64% of participants reached the target blood pressure goal of 130/80 mm Hg) when barber-initiated health promotion was coupled with follow-up care provided by pharmacists in the barbershop.

Patrons were encouraged by their barbers to be counseled by specialty-trained pharmacists in the barbershop who prescribed drug therapy under a collaborative practice agreement with the patrons' physicians. In the less-effective “control” arm (12% of participants reached target blood pressure), barbershop patrons were encouraged by their barbers to engage in healthy lifestyle behaviors and make appointments with their physicians. In light of pharmacists often being considered as the most trusted^19^ and accessible^20^ experts of drug therapy (including the fact that counseling is often provided without appointments or copays), these findings illustrate how pharmacist-led interventions can improve health outcomes for disproportionately affected groups through community-based approaches in nontraditional settings.

Before pharmacist-led interventions can be implemented in Black, Indigenous, and people of color communities on a broad scale, it is important to understand if these trial results are generalizable to real-world settings and sustainable long-term. Indeed, barbershop patrons were provided with incentives for participation, including free haircuts and cash to cover pharmacy expenses (such as transportation and generic drugs). Furthermore, for participants without a primary care physician, a designated community physician was available to sign the collaborative agreement with the pharmacist. Moreover, results were only available for a follow-up of 6 months. These factors likely enhanced participation (95% of the participants remained in the study through the end of follow-up), but may not be feasible for broad implementation.

Evidence suggests that patient perspective is a strong predictor of adherence to a healthy lifestyle modification plan.^21,22^ Thus, a better understanding of how potential future participants view health care services in nontraditional settings, including any barriers to these services is a necessary step to ascertain the likelihood for success of these interventions in the real world.

Our study objective was to explore the perspectives and preferences of Black male barbershop patrons regarding health care-provided services in nontraditional settings. Results inform the potential utility of innovative pharmacy practice services to improve outcomes in groups that have been historically marginalized.

## Methods

### Study population and setting

Participants were engaged and recruited at a single Black-owned barbershop located in the San Francisco Bay Area of the United States from March 2022 through April 2022. The study was conducted in collaboration with (and with full support from) the barbershop owner and barbers.^23^ Patrons aged 18 years or older in the barbershop to get a haircut or for any other reason were eligible to participate in the study. Although doctoral-level pharmacists and pharmacy students helped to conduct the study, there was no pharmacy involved (pharmacists and pharmacy students engaged participants within the barbershop). Participants provided informed consent for study participation. The study was exempt by the University of California San Francisco (UCSF) Institutional Review Board.

### Survey

The study design of this investigation was a cross-sectional, community-based, qualitative, survey. We developed a 14-question online survey (Qualtrics, 2022) to obtain individual attitudes and preference toward the idea of receiving health services in nontraditional clinical settings. Race, ethnicity, and other demographic information were self-identified by survey participants. Sample questions were piloted to a small group of participants and revised by all research team members during survey development. Piloted responses were not included in the final data collection. The list of verbatim survey questions are as follows:

(1) Acknowledgment to participate in survey (Agree, Disagree)
(2) Age
(3) Sex (female, male, other)
(4) Race (American Indian/Alaska Native, Asian, Native Hawaiian/Other Pacific Islander, Black/African American, White, Other)
(5) Ethnicity (Hispanic/Latino, not Hispanic/Latino, Unknown ethnicity)
(6) How many times a year do you see your primary care provider? (1–2, 3–5, 5–7, 7+)
(7) Do you wish you saw a primary provider more often? (yes, no)
(8) Follow-up to the question above: please elaborate on why or why not?
(9) The following barriers impact your ability to receive health care (e.g., visits with a primary care provider, follow-ups for chronic disease management, communication with a health care provider): (Likert Scale: Transportation, Cost of visit, Cost of medication, Culture, Education level, Relationship with provider, Insurance coverage, Other)
(10) You would prefer to get your health care (e.g., visits with a primary care provider, follow-ups for chronic disease management, communication with a health care provider) at: (Likert Scale: Church, Grocery Store, Traditional Clinic, Barbershop, At Home, Pharmacy, Other)
(11) If you selected “Agree” or “Strongly Agree” for any of the above options, please briefly explain why for each: (free response)
(12) What concerns do you have about receiving care in a nontraditional setting (e.g., barbershop, church, grocery store): (free response)
(13) Did you participate in our optional Blood Pressure Screening service today? (yes, no)
(14) Would you like assistance in accessing care? Please check all that apply: (Finding a Primary Care Provider, Obtaining Health Insurance, Help with medication costs, help with office visits costs, other, no thank you)

### Community engagement, recruitment, and survey administration

Potential participants were asked by their barber or the pharmacy team if they would like to have their blood pressure taken in a private space in the back of the barbershop. Barbers had previously been trained as health coaches for patrons to promote healthy lifestyle changes and optional blood pressure screenings. Patrons who took part in the optional blood pressure service received education from on-site pharmacists or pharmacist interns about what hypertension is, why it is important, and potential lifestyle changes to prevent or treat it. Regardless of receiving a blood pressure screening, patrons were asked if they would like to take part in the current survey study. If patrons agreed to take the survey, the pharmacy study team distributed the survey electronically through a quick response code or manually with a hardcopy document.

Surveys were taken in-person by the respondents on a mobile device or using paper and pencil (on the hardcopy) with the study team present. Members of the study team were physically available at the time of survey completion for any questions and concerns from study participants.

The aforementioned training of barbers into health coaches was conducted by the Bay Area Cut Hypertension Program with support from the UCSF Center for Excellence in Primary Care. As stated, the blood pressure screenings were not a requirement for study participation, but were useful as a conversation starter to introduce the survey to the barbershop patrons.

### Data analysis

We generated descriptive statistics from the survey responses to complete the main objective of the study. Likert scale responses were displayed as categorical data. Our primary analysis for the current study was conducted from the survey results of the self-reported non-Hispanic Black male study participants. Participants were considered non-Hispanic if they skipped, selected “unknown,” or selected “non-Hispanic” for the “non-Hispanic versus Hispanic” ethnicity category. We considered barbershops, churches, and grocery stores to be nontraditional health care settings. Clinics, pharmacies, and home care were considered to be traditional settings.

## Results

### Characteristics of study population

Of the 33 patrons who were offered the survey, only 2 declined (Table 1). From the 31 enrolled participants, 26 self-identified as male and 5 as female. The age range was 21–78 years with a mean age of 38 years. The majority of survey participants self-identified as non-Hispanic Black males (*n*=17). All females in the study self-identified as non-Hispanic Black. Consistent with the primary objective of the study, reported results pertain to the 17 Black males unless otherwise stated.

**Table 1.** Survey Participant Demographics

	***n*** =31
Age^a^	
21–40	20
41–65	3
65+	6
Race/Ethnicity	
Hispanic	3
Non-Hispanic White	1
Non-Hispanic Asian	4
Non-Hispanic Black	23
Sex	
Male	26
Female	5

^a^ Two participants did not report age in the survey.

### Desire to participate in blood pressure screening and perceived need for health care

Of the 17 Black men participating in the study, 71% opted to receive a free blood pressure screening from a pharmacist or pharmacist intern. Only 29% reported visiting their provider >2 times per year. In addition, 77% stated that they did not wish to see a primary care provider more frequently than their current frequency of use. For respondents who did not desire increased health care interactions, when asked to elaborate on why in free form response, many reported not having an active problem necessitating a primary care visit. Among those who desired more interactions, two participants listed maintaining their health and engaging in preventative care as reasons for why.

### Preferences for receiving health care

**FIG. 1.**
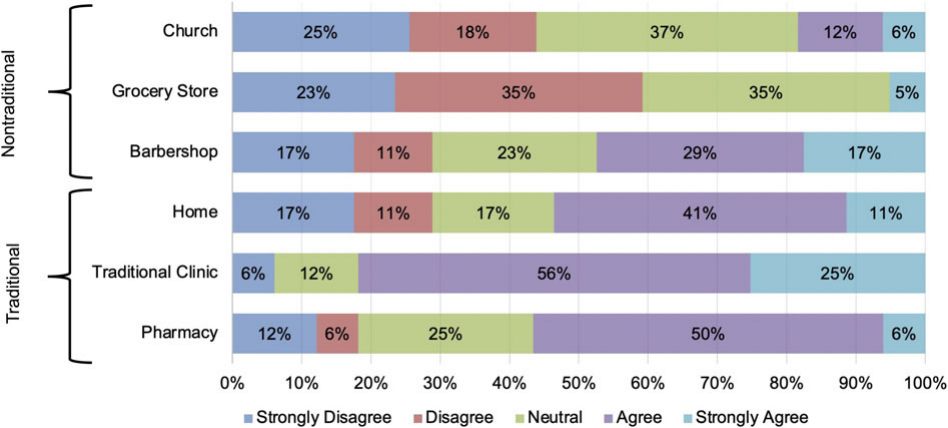
Preferences for receiving health care for self-identified non-Hispanic Black men (*n*=17).

The majority of participants (81%) agreed or strongly agreed that they would like to receive services from a traditional clinic setting as the most preferred health care setting (Fig. 1). The pharmacy (56% agreed or strongly agreed) and receiving care in the patient's own home (53% agreed or strongly agreed) were the next most preferred location to receive health care. A minority of participants agreed or strongly agreed that receiving health care at the barbershop (47%), church (19%), or grocery store (6%) were preferred settings. Thus, participants overall preferred traditional health care settings compared to nontraditional settings.

### Barriers and concerns to receiving health care

**FIG. 2.**
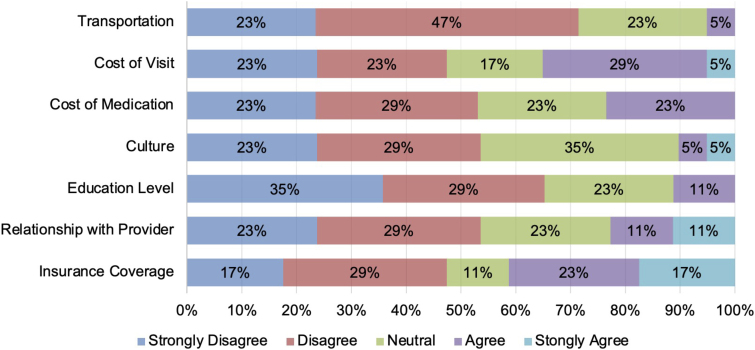
Barriers to receiving health care for self-identified non-Hispanic Black men (*n*=17).

Participants reported numerous barriers to receiving health care (in both traditional and nontraditional settings), but they varied greatly. Among all available options, there were none in which the majority of participants agreed or strongly agreed that it was a barrier to receiving health care (Fig. 2). However, the majority (53%) of respondents agreed or strongly agreed that at least one of the options was a barrier to receiving health care. Insurance coverage was the option most likely to receive an “agree” or “strongly agree” (41%) as a barrier to receiving health care. Cost of a visit was also a major barrier (35% of participants agreed or strongly agreed).

Options least reported as barriers were transportation (only 5% agreed or strongly agreed that this was a barrier), culture (10% agreed or strongly agreed), and education level (11% agreed or strongly agreed). Among free responses, participants (*n*=4) also cited convenience as a barrier to receiving health care. For example, one respondent stated that they “…do find that there are barriers to […] consistently seeking primary care. Cost is one concern, but it's also just a big convoluted […] process to find a [primary care provider].”

When asked about potential concerns with receiving health care from a nontraditional setting (e.g., barbershop, church, grocery store) in the free form response, participants often cited privacy (*n*=7), quality, and sanitation. With regard to privacy as a concern, one respondent noted that the “church and barbershop are filled with familiar faces.” Another individual brought up “…a small question of patient dignity and potential HIPAA [Health Insurance Portability and Accountability Act] violations.” With regard to quality, one participant remarked that in contrast to nontraditional sites of care, traditional settings “have trained professionals” and are built explicitly for patient care.

Another noted the following: “I prefer to receive medical care in a medical environment not in a different location that has been repurposed.” Likewise, a third notable response was that “[nontraditional sites of care] aren't purpose-built. From a design perspective, care will be suboptimal in any location not expressly built to provide care.” Among the multiple comments related to potential sanitation concerns of receiving care in a nontraditional health care setting such as a barbershop, one response of note was that “It's just not as clean as it should be.”

## Discussion

Despite great advances related to the ways we detect, prevent, diagnose, and treat disease in the United States, Black people continue to have a higher risk of poor health-related outcomes than their White counterparts.^23^ Enduring structures that limit the accessibility of key health care services and educational resources are the sources of these health disparities.^9^

In this study, we collaborated with a Black-owned barbershop of the San Francisco Bay Area to engage patrons and gather their thoughts regarding receiving health services for primary care in nontraditional settings. A major strength of this study design was the ability to gather rich, qualitative data from a relatively small sample size. In addition, our study participants tended to be younger adults (30–50 years of age), the age at which initial screenings for cardiovascular chronic diseases are recommended.^23^ Results may guide future pharmacy-led interventions in nontraditional, ambulatory settings for preventive services. Ultimately, these creative interventions have the potential to improve health care outcomes for groups that have not received equitable health care in the United States by breaking down long-standing barriers in the health system.

The majority of participants reported barriers to receiving health care, but they varied greatly by individual. The most common perceived barriers to health care services were related to affordability (poor insurance coverage, cost of an appointment, cost of medication). This is not an uncommon finding among prior reports. The uninsured rate is the second-highest in Black Americans compared to other race/ethnicity groups.^24,25^ In addition, the high cost of medications has previously been cited as a critical barrier for access to interventions for diabetes, cardiovascular disease, and hypertension.^26,27^ Even among those with adequate insurance coverage in our study, participants described difficulty in navigating the system to select the best coverage options and in-network providers, similar to past findings.^28^ As one study patron had noted, finding a primary care provider was an overall “convoluted” process.

Second to cost-related barriers, participants in the current study reported the lack of a close relationship with their provider as a significant barrier to receiving health care. This is also not a surprising finding as lack of trust has been observed as a factor preventing access to health care among historically minoritized groups.^29,30^ One source of this barrier is the lack of representation of Black people among health care professionals. People who identify as Black make up 12% of the working age population,^31^ but only 5% of the physician workforce.^32^ Both affordability, patient-provider relationships, and lack of representation as barriers to health care fall under the umbrella of structural racism in this country.^31–33^

The evidence demonstrating the promise of nontraditional health care settings as a more culturally sensitive, accessible, and cost-effective alternative to traditional settings has accumulated in recent years. For example, the Church/Community Health Awareness and Monitoring Program showed success from training over 500 church volunteers from ∼100 churches in Baltimore, Maryland, to monitor blood pressure and make appropriate follow-up health care referrals as needed.^34^

Moreover, the New Orleans-based Healthy Heart Community Prevention Project recruited pastors from five churches who provided health sermons to their congregation on reducing cardiovascular disease risk factors and practicing specific healthy lifestyle behaviors.^14^ Project Joy collaborated with three churches for a 1-year program to maintain nutrition and physical activity strategies for Black women^35^ while another church-based outreach program prepared registered nurses as “Church Health Educators” to use a hypertension education and support program for Black congregation members.^36^ Other venues for health care have included the workplace,^15,16^ farms,^17^ and firehouses.^18^

In addition, pharmacist-led interventions for medication-related services previously performed by other health care professionals have also demonstrated efficacy, safety, and cost benefits.^37,38^ This includes the findings from a follow-up analysis of LABBPS, which demonstrated the cost-effectiveness of pharmacist-led interventions in Black-owned barbershops.^39^ Altogether, these data demonstrate the benefits of using nontraditional health care settings or pharmacist-led interventions and provide compelling rationale to scale these models of care delivery in communities at larger scale.

These novel community-based approaches have the potential to attenuate the strongest barriers to health care (access and cost) reported by the participants of the current study. Interestingly, our overall results suggest a substantial level of skepticism among participants with receiving care in nontraditional settings. Barbershop patrons expressed doubt toward the authenticity, quality (in terms of privacy protection, sanitation, and professionalism), and legitimacy of these services. One potential reason for this finding is simply the lack of familiarity. As one respondent articulated, they prefer a traditional clinic “because it's the status quo.” Therefore, the success of these nontraditional health care establishments for improving outcomes in a real-world setting may be dependent on increased public acceptance. Enhanced awareness that dispels misconceptions and promotes the benefits may be necessary before these interventions are implemented outside of clinical trials and small pilots.

Our study has limitations. First, this study is a small, descriptive study underpowered to detect statistical differences. However, our investigation is intended to be an exploratory pilot to assess perceptions and attitude in a qualitative manner. Second, our study was conducted in a single setting, which limits generalizability. However, our pilot paves the way for future studies in multiple institutions. Finally, although our survey was piloted and revised by independent research personnel, further validation is required to ensure that we accurately assessed attitudes and preference of receiving health services in nontraditional clinical settings.

## Conclusion

We report the perspectives and preferences of Black male barbershop patrons as it pertains to receiving health care in nontraditional settings. Granted that respondents expressed noteworthy reservations and distrust regarding services within these innovative models of care, more conversations may be necessary to increase acceptability. Future studies should 1) assess participant perceptions in larger study populations across multiple institutions and 2) investigate the effectiveness of educational interventions to enhance awareness for nontraditional health care settings.

## Abbreviation Used

UCSF: University of California San Francisco

## References

[1] Menke A, Rust KF, Fradkin J, et al. Associations between trends in race/ethnicity, aging, and body mass index with diabetes prevalence in the United States: A series of cross-sectional studies. Ann Intern Med 2014;161(5):328–335; doi: 10.7326/M14-0286 25178569

[2] Community Screening for Pre-Diabetes and Diabetes Using HbA1c Levels in High Risk African Americans and Latinos—PMC. Available from: https://www.ncbi.nlm.nih.gov/pmc/articles/PMC4287403/ [Last accessed: May 29, 2022]. PMC4287403 24804366

[3] Coronary Heart Disease, Myocardial Infarction, and Stroke—A Public Health Issue | CDC. 2019. Available from: https://www.cdc.gov/aging/publications/coronary-heart-disease-brief.html [Last accessed: May 29, 2022].

[4] Carnethon MR, Pu J, Howard G, et al. American Heart Association Council on Epidemiology and Prevention; Council on Cardiovascular Disease in the Young; Council on Cardiovascular and Stroke Nursing; Council on Clinical Cardiology; Council on Functional Genomics and Translational Biology; and Stroke Council. Cardiovascular health in African Americans: A scientific statement from the American Heart Association. Circulation 2017;136(21):e393–e423; doi: 10.1161/CIR.0000000000000534 29061565

[5] Cutler JA, Sorlie PD, Wolz M, et al. Trends in hypertension prevalence, awareness, treatment, and control rates in United States adults between 1988–1994 and 1999–2004. Hypertension 2008;52(5):818–827; doi: 10.1161/HYPERTENSIONAHA.108.113357 18852389

[6] Carey RM, Whelton PK; 2017 ACC/AHA Hypertension Guideline Writing Committee. Prevention, detection, evaluation, and management of high blood pressure in adults: Synopsis of the 2017 American College of Cardiology/American Heart Association hypertension guideline. Ann Intern Med 2018;168(5):351–358; doi: 10.7326/M17-3203 29357392

[7] Ashman JJ, Rui P, Schappert SM, et al. Characteristics of visits to primary care physicians by adults diagnosed with hypertension. Natl Health Stat Report 2017;106:1–14. PMID: 29155688. 29155688

[8] Odlum M, Moise N, Kronish IM, et al. Trends in poor health indicators among black and hispanic middle-aged and older adults in the United States, 1999–2018. JAMA Netw Open 2020;3(11):e2025134; doi: 10.1001/jamanetworkopen.2020.25134 33175177 PMC7658737

[9] Williams DR, Rucker TD. Understanding and addressing racial disparities in health care. Health Care Financ Rev 2000;21(4):75–90. 11481746 PMC4194634

[10] Rader F, Elashoff RM, Niknezhad S, et al. Differential treatment of hypertension by primary care providers and hypertension specialists in a barber-based intervention trial to control hypertension in Black men. Am J Cardiol 2013;112(9):1421–1426; doi: 10.1016/j.amjcard.2013.07.004 23978276 PMC3800500

[11] Hess PL, Reingold JS, Jones J, et al. Barbershops as hypertension detection, referral, and follow-up centers for black men. Hypertension 2007;49(5):1040–1046; doi: 10.1161/HYPERTENSIONAHA.106.080432 17404187

[12] Victor RG, Ravenell JE, Freeman A, et al. Effectiveness of a barber-based intervention for improving hypertension control in black men: The BARBER-1 study: A cluster randomized trial. Arch Intern Med 2011;171(4):342–350; doi: 10.1001/archinternmed.2010.390 20975012 PMC3365537

[13] Schoenthaler AM, Lancaster KJ, Chaplin W, et al. Cluster randomized clinical trial of FAITH (Faith-Based Approaches in the Treatment of Hypertension) in blacks. Circ Cardiovasc Qual Outcomes 2018;11(10):e004691; doi: 10.1161/CIRCOUTCOMES.118.004691 30354579

[14] Ferdinand KC. The Healthy Heart Community Prevention Project: A model for primary cardiovascular risk reduction in the African-American population. J Natl Med Assoc 1995;87(8 Suppl):638–641. 7674364 PMC2607934

[15] Pelletier KR. A review and analysis of the clinical and cost-effectiveness studies of comprehensive health promotion and disease management programs at the worksite: Update VIII 2008 to 2010. J Occup Environ Med 2011;53(11):1310–1331; doi: 10.1097/JOM.0b013e3182337748 22015548

[16] Clemow LP, Pickering TG, Davidson KW, et al. Stress management in the workplace for employees with hypertension: A randomized controlled trial. Transl Behav Med 2018;8(5):761–770; doi: 10.1093/tbm/iby018 30202927 PMC6128963

[17] Rajjo T, Mohammed K, Rho J, et al. On-the-farm cardiovascular risk screening among migrant agricultural workers in Southeast Minnesota: A pilot prospective study. BMJ Open 2018;8(7):e019547; doi: 10.1136/bmjopen-2017-019547 PMC6067365 30061429

[18] Frattaroli S, Pollack KM, Bailey M, et al. Working inside the firehouse: Developing a participant-driven intervention to enhance health-promoting behaviors. Health Promot Pract 2013;14(3):451–458; doi: 10.1177/1524839912461150 23091304

[19] Gregory PAM, Austin Z. Understanding the psychology of trust between patients and their community pharmacists. Can Pharm J (Ott) 2021;154(2):120–128; doi: 10.1177/1715163521989760 33868523 PMC8020281

[20] Manolakis PG, Skelton JB. Pharmacists' contributions to primary care in the United States collaborating to address unmet patient care needs: The emerging role for pharmacists to address the shortage of primary care providers. Am J Pharm Educ 2010;74(10):S7; doi: 10.5688/aj7410s7 21436916 PMC3058447

[21] Mitchell AJ. Adherence behaviour with psychotropic medication is a form of self-medication. Med Hypotheses 2007;68(1):12–21; doi: 10.1016/j.mehy.2006.07.005 16996228

[22] Petit G, Berra E, Georges CMG, et al. Impact of psychological profile on drug adherence and drug resistance in patients with apparently treatment-resistant hypertension. Blood Press 2018;27(6):358–367; doi: 10.1080/08037051.2018.1476058 29952236

[23] Arnett DK, Blumenthal RS, Albert MA, et al. 2019 ACC/AHA guideline on the primary prevention of cardiovascular disease: A report of the American College of Cardiology/American Heart Association Task Force on clinical practice guidelines. Circulation 2019;140(11):e596–e646; doi: 10.1161/CIR.0000000000000678. Erratum in: Circulation 2019;140:e649–e650. Erratum in: Circulation 2020;141(4):e60. Erratum in: Circulation 2020;140:e774.30879355

[24] Health Insurance Coverage and Access to Care Among Black Americans: Recent Trends and Key Challenges. ASPE. Available from: https://www.aspe.hhs.gov/reports/health-insurance-coverage-access-care-among-black-americans [Last accessed: May 29, 2022].

[25] Keisler-Starkey K, Bunch LN. U.S. Census Bureau Current Population Reports, P60-271, Health Insurance Coverage in the United States: 2019, U.S. Government Publishing Office, Washington, DC, 2020.

[26] Kennedy J, Tuleu I, Mackay K. Unfilled prescriptions of Medicare beneficiaries: Prevalence, reasons, and types of medicines prescribed. J Manag Care Spec Pharm 2020;26(8):935–942; doi: 10.18553/jmcp.2020.26.8.935 32715958 PMC10391240

[27] Konstantinou P, Kassianos AP, Georgiou G, et al. Barriers, facilitators, and interventions for medication adherence across chronic conditions with the highest non-adherence rates: A scoping review with recommendations for intervention development. Transl Behav Med 2020;10(6):1390–1398; doi: 10.1093/tbm/ibaa118 33231691

[28] Ali NM, Combs RM, Muvuka B, et al. Addressing health insurance literacy gaps in an urban African American population: A qualitative study. J Community Health 2018;43(6):1208–1216; doi: 10.1007/s10900-018-0541-x 29926271

[29] Murray TM. Trust in African Americans' healthcare experiences. Nurs Forum 2015;50(4):285–292; doi: 10.1111/nuf.12120 25612227

[30] Pugh M Jr., Perrin PB, Rybarczyk B, et al. Racism, mental health, healthcare provider trust, and medication adherence among black patients in safety-net primary care. J Clin Psychol Med Settings 2021;28(1):181–190; doi: 10.1007/s10880-020-09702-y 32008136

[31] Halbert CH, Armstrong K, Gandy OH Jr, et al. Racial differences in trust in health care providers. Arch Intern Med 2006;166(8):896–901; doi: 10.1001/archinte.166.8.896 16636216

[32] Labor force characteristics by race and ethnicity, 2018: BLS Reports: U.S. Bureau of Labor Statistics. Available from: https://www.bls.gov/opub/reports/race-and-ethnicity/2018/home.htm [Last accessed: May 29, 2022].

[33] Figure 18. Percentage of all active physicians by race/ethnicity, 2018. AAMC. Available from: https://www.aamc.org/data-reports/workforce/interactive-data/figure-18-percentage-all-active-physicians-race/ethnicity-2018 [Last accessed: May 29, 2022].

[34] Kong BW, Miller JM, Smoot RT. Churches as high blood pressure control centers. J Natl Med Assoc 1982;74(9):920–923.

[35] Yanek LR, Becker DM, Moy TF, et al. Project Joy: Faith based cardiovascular health promotion for African American women. Public Health Rep 2001;116(Suppl 1):68–81; doi: 10.1093/phr/116.S1.68 11889276 PMC1913665

[36] Smith ED, Merritt SL, Patel MK. Church-based education: An outreach program for African Americans with hypertension. Ethn Health 1997;2(3):243–253; doi: 10.1080/13557858.1997.9961832 9426988

[37] Houle SK, Chuck AW, McAlister FA, et al. Effect of a pharmacist-managed hypertension program on health system costs: An evaluation of the Study of Cardiovascular Risk Intervention by Pharmacists-Hypertension (SCRIP-HTN). Pharmacotherapy 2012;32(6):527–537; doi: 10.1002/j.1875-9114.2012.01097.x 22552863

[38] Jay JS, Ijioma SC, Holdford DA, et al. The cost-effectiveness of pharmacist-physician collaborative care models vs usual care on time in target systolic blood pressure range in patients with hypertension: A payer perspective. J Manag Care Spec Pharm 2021;27(12):1680–1690; doi: 10.18553/jmcp.2021.27.12.1680 34818090 PMC10390951

[39] Bryant KB, Moran AE, Kazi DS, et al. Cost-effectiveness of hypertension treatment by pharmacists in black barbershops. Circulation 2021;143(24):2384–2394; doi: 10.1161/CIRCULATIONAHA.120.051683 33855861 PMC8206005

